# SUVmax/THKmax as a Biomarker for Distinguishing Advanced Gastric Carcinoma from Primary Gastric Lymphoma

**DOI:** 10.1371/journal.pone.0050914

**Published:** 2012-12-04

**Authors:** Liping Fu, Hongming Li, Hui Wang, Baixuan Xu, Yong Fan, Jiahe Tian

**Affiliations:** 1 Department of Nuclear Medicine, General Hospital of the Chinese People’s Liberation Army and Military Medical Postgraduate College, Beijing, China; 2 National Laboratory of Pattern Recognition, Institute of Automation, Chinese Academy of Sciences, Beijing, China; Banner Alzheimer's Institute, United States of America

## Abstract

**Background:**

Gastric carcinoma and primary gastric lymphoma (PGL) are the two most common malignancies in stomach. The purpose of this study was to screen and validate a biomarker of ^18^F-fluorodeoxyglucose positron emission tomography/computed tomography (^18^F-FDG PET/CT) for distinguishing advanced gastric carcinoma (AGC) from PGL for clinical applications.

**Methodology/Principal Findings:**

We reviewed PET/CT scans collected from January 2008 to April 2012 of 69 AGC and 38 PGL (14 low-grade mucosa-associated lymphoid tissue [MALT], 24 non-MALT aggressive non-Hodgkin lymphoma [ANHL]) with a focus on FDG intensity (maximum standardized uptake value [SUVmax]) of primary lesions and its CT-detected abnormalities, including maximal gastrointestinal wall thickness (THKmax) and mucosal ulcerations. Gastric FDG uptake was found in 69 (100%) patients with AGC and 36 (95%, 12 MALT vs. 24 ANHL)with PGL. The presence of CT-detected abnormalities of AGC and PGL were 97% (67/69) and 89% (12 MALT vs. 22 ANHL), respectively. After controlling for THKmax, SUVmax was higher with ANHL than AGC (17.10±8.08 vs. 9.65±5.24, *p*<0.05) and MALT (6.20±3.60, *p*<0.05). THKmax did not differ among MALT, ANHL and AGC. Mucosal ulceration was more common with AGC (n = 9) than PGL (n = 2),but the difference was not statistically significant (*p*>0.05). Cross-validation analysis showed that for distinguishing ANHL from AGC, the classifier with SUVmax as a feature achieved a correct classification rate of 81% with thresholds 13.40±1.12 and the classifier with SUVmax/THKmax as a feature achieved a correct classification rate of 83% with thresholds 7.51±0.63.

**Conclusions/Significance:**

SUVmax/THKmax may be as a promising biomarker of FDG-PET/CT for distinguishing ANHL from AGC. Structural CT abnormalities alone may not be reliable but can help with PET assessment of gastric malignancies. ^18^F-FDG PET/CT have potential for distinguishing AGC from PGL at the individual level.

## Introduction

Gastric carcinoma (GC) and primary gastric lymphoma (PGL) are the 2 most commonly encountered malignant entities in the stomach. Despite its declining incidence, GC still ranks fourth in incidence among cancers worldwide, with high disease-related mortality [1]. Although neoadjuvant treatment strategies with chemo- and/or radiotherapy are typically used, radical resection, including adequate lymphadenectomy, remains the major treatment [2]. Primary gastric non-Hodgkin’s lymphoma accounts for less than 15% of gastric malignancies and 2% of lymphomas, and the stomach is the most common site of extra-nodal involvement in non-Hodgkin’s lymphoma [3], [4]. Although there has been a debate on the best therapeutic strategy for PGL, it has been generally believed that for localized lesion(s) in the early stages (stage I E & II E), surgery is adequate and of clinical benefit. However, for advanced stages (stage III E, IV E, or unresectable II E), chemotherapy and/or radiation might be the best option, with surgery reserved for cytoreduction or chemo/radiotherapy-induced complications [5].

The prognosis for the 2 malignancies is difficult because prognosis largely depends on stage and histology and varies by treatments. However, with surgery alone, the 5-year survival rate is lower for GC than PGL in the early stage (70% vs. 82%–95%, stage I E) and advanced stage (6%–7% vs. 27%–31%, stage IV E) [6]–[8]. Thus, differences in the management and prognosis for GC and PGL highlight the importance of accurate detection and differentiation of GC and PGL.

Both GC and PGL share non-specific clinical manifestations, such as adnominal pain and dyspepsia, as well as the similar morphological characters, such as gastric ulcers and irregular thickness of gastric wall. Although endoscopy of the stomach, followed by biopsy, can provide valuable information for diagnosis, the diversity of endoscopy findings (malignant ulcer and mucosal erosion) and the specific biological behavior (deep infiltration beneath the mucous layer) with PGL could lead to false-negative endoscopy/biopsy results [8]. In China, the preoperative misdiagnosis rate for PGL is up to 80%, much higher than that of GC [9].

For better prognosis and management of GC and PGL patients, preoperative imaging might be useful. A growing body of literature supports the use of ^18^F-fluorodeoxyglucose positron emission tomography/computed tomography (FDG-PET/CT) for imaging GC and PGL [10]–[13]. However, controversial results were frequently reported, which might be due in part to different FDG avidity of subtypes of GC and PGL [14], [15].

## Methods

### Objectives

We aimed to screen and validate a valid biomarker using ^18^F-FDG PET/CT for distinguishing advanced GC (AGC) from PGL for clinical applications.

### Participants

**Figure 1:**
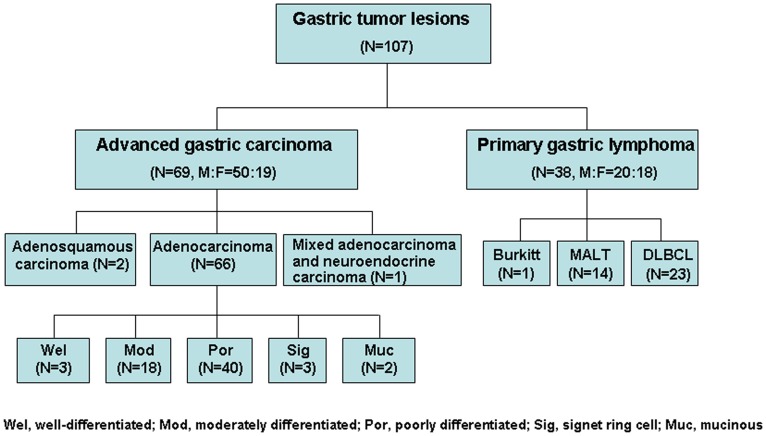
Histological composition of study enrollment.

We reviewed 107 consecutive patients suffering from PGL and AGC from January 2008 to April 2012. Inclusion criteria were (1) newly pathologically diagnosed PGL and AGC before any clinical treatment and (2) FDG-PET/CT scanning and surgical resection or endoscopy biopsy completed within 2 weeks. The patient cohort included 69 AGC (male: female ratio 50∶19) and 38 PGL (14 low-grade mucosa-associated lymphoid tissue [MALT], 24 aggressive non-Hodgkin’s lymphoma [ANHL]; male: female ratio 20∶18). The mean age of patients with AGC and PGL was 63±13 (range 39–84) and 52±18 (range 15–89) years, respectively. The histological results of gastric tumors are shown in Figure 1.

### FDG-PET/CT Imaging

Patients were instructed to fast for 4–6 h, and blood samples were taken before FDG injection to ensure that blood glucose level was <7.0 mmol/l (126 mg/dl). The injected dose of FDG ranged from 296–444 MBq (8–12 mCi). After injection, patients remained lying comfortably in a quiet, dimly illuminated room for 50–60 min. They were required to drink 600 ml water before image acquisition to distend the proximal part of the stomach.

Each patient underwent whole-body FDG-PET scanning with PET/CT systems from GE (Discovery DVCT, General Electric Healthcare, USA) or SIEMENS (Biography Truepoint 64, Siemens Healthcare, Germany) in a random order. Both PET/CT scanners were composed of a dedicated PET scanner and a multi-slice CT, and the detection and quantification parameters were normalized regularly by a phantom as part of the quality control practice in our center. A routine imaging protocol involved initial CT acquisition, then PET scanning. Acquisition parameters for CT were 120 kV, 100 mA, and 4-mm slice thickness. PET scans were acquired in a series of 15- to 21-cm coverage per bed to cover the trunk region from skull base to the upper femur. PET acquisition was performed in three-dimensional mode with a matrix of 128×128. PET data were reconstructed by an iterative method (VUE point for GE and True X for SIEMENS). Data from CT were used for attenuation correction of PET emission data and for fusion of attenuation-corrected PET images with corresponding CT images.

After completion of the scanning, CT, FDG-PET and fused PET/CT images were reviewed in transverse, coronal and sagittal planes and in a maximum-intensity-projection 3-D cine mode. Semi-quantitative analysis involved mainly transverse and coronal images with the commercial software provided with the workstations (Xeleris of GE and Syngo of SIEMENS).

### Image Interpretation

A team of 2 experienced nuclear medicine physicians and a radiologist interpreted all FDG-PET/CT images with knowledge of the clinical history but not the results of pathology and gastroscopy findings for patients. Three image readers used the following interpretation criteria to achieve consensus in diagnosis.

PET assessments were performed in the presence of corresponding CT images and further clinical conformation. The presence and intensity of gastric FDG uptake were assessed visually and semi-quantitatively. Results were positive if the uptake was higher than the hepatic uptake. Semi-quantitative measurements of gastric FDG uptake were expressed as the maximum standard uptake value (SUVmax). CT images were assessed by the presence of mucosal ulcerations and maximal gastrointestinal wall thickness (THKmax).

We collected data on the presence and intensity of FDG uptake (SUVmax) in gastric, as well as CT-observed abnormalities (THKmax and mucosal ulceration) of gastric lesions into a dataset ready for further analysis.

### Ethics

The study was implemented at the General Hospital of the Chinese People's Liberation Army and Military Medical Postgraduate College. All procedures for FDG-PET/CT examination were approved by the Medical Ethics Committee of Chinese PLA general hospital, and all participants or an appropriate representative signed informed consent forms after a complete written and verbal description of the study.

### Parameter Characterization

To screen potential diagnostic parameter(s) with proper threshold to distinguish AGC and PGL at the individual level, we used a pattern classification technique. Briefly, a classifier was learned on the basis of certain classification criteria from training samples with different features (such as SUVmax, THKmax value and other clinical variables), then the classifier was used to provide a class prediction for new samples. The predication capability, i.e., diagnostic accuracy, of the classifier was evaluated in light of the pathological results. In the current study, we used the Fisher linear discriminant [16]–[18] by prtools [19] to distinguish ANHL from AGC only, because the number of MALT cases was relatively small. Except for 2 cases each of AGC and ANHL with the presence of FDG uptake but not CT abnormalities of the gastric wall, 67 AGC and 22 ANHL were used for further analysis. Fisher linear discriminant projects the *d*-dimensional feature vector into one dimension to maximize the class-separation. For a two-class problem, the class-separation is defined as
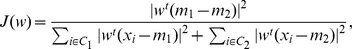
where w is the projection coefficient, and
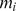
is the mean of feature vectors of class i, and
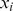
is the feature vector of sample i in the training dataset. The numerator and denominator represent the inter-class separation and the intra-class variance after projection, respectively. The objective is to partition the training samples into classes with large inter-class separation and small intra-class variance. With the optimal w achieved from training samples, the classification is finally implemented as



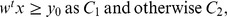
where x is a feature vector to be classified and 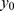
is a threshold value. In our study, 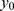
was computed by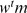, where
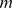
was the mean of all the training feature vectors. Different combinations of features were used including (a) SUVmax and THKmax, (b) SUVmax only, (c) SUVmax/THKmax and THKmax, (d) SUVmax/THKmax only. To avoid the imbalanced training sample problem, i.e., one class is represented by a large number of instances while the other is represented by a small number of instances, 11 (half of the total of the small class) ANHL subjects and 11 AGC subjects were randomly selected and used for training while the left subjects for testing. This procedure was repeated 100 times, and the mean classification error and mean classification threshold value of individual features were computed.

Permutation test technique [20] was used to evaluate the statistical significance of the classification performance. The null hypothesis assumes that the two classes considered are indistinguishable with respect to the statistic related to the classification performance, and the alternative hypothesis is that a classifier with small expected error could be obtained. Under the null hypothesis all the training datasets generated through permutations are equally likely to be observed, yielding the estimates of the selected statistic for the empirical cumulative distribution. Let
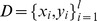
be the original dataset and *CF* be the configuration of training and testing samples used in each run of the 100-repetition procedure described above, the permutation test for classification can be summarized as following:

1. Repeat *M* times:
  - Permute the labels of the all samples
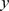
randomly, forming a permuted dataset
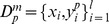
.
  - Implement the classification with
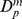
under *CF*, and compute the corresponding statistical value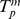.
2. Construct an empirical cumulative distribution

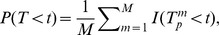
where *I(x)* is a function that equals to 1 if *x* is true and 0 otherwise.

1. Implement the classification with the original dataset *D* under *CF* and compute its statistic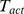. Its corresponding *p*-value under the empirical distribution *P* is calculated as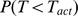.

It is worth noting that the permutation was repeated 500 times (*M* = 500) for each run under its corresponding *CF* for assessing the classification performance. Classification error, sensitivity, and specificity were the statistics considered, respectively, and *p*-values associated with these statistics were calculated as:
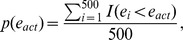


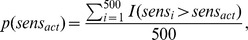


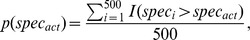
where
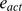
is the classification error,
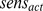
is the sensitivity, and
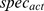
is the specificity.

### Statistical Analysis

Since tumor volume and tumor thickness have been previously found positively correlated with SUVmax in MALT [21], we used an ANCOVA test with THKmax used as a control covariate and SUVmax as a covariate of interest to assess FDG uptake among 3 sub-groups: ANHL, MALT and AGC. One-way ANOVA was used to evaluate SUVmax and THKmax for gastric lesions. Chi-square test was used to analyze differences in ulceration between PGL and AGC. Pearson correlation analysis was used to test the correlation of THKmax and SUVmax. All statistical tests involved use of SPSS 13.0 (SPSS Inc., Chicago, IL). *P*<0.05 was considered statistically significant.

## Results

### FDG Uptake in Gastric Lesions

The presence of gastric FDG uptake and SUVmax are summarized in Table 1. ANOVA revealed that SUVmax was higher in patients with ANHL than MALT and AGC (*F* = 18.34, *p*<0.001); ANCOVA revealed the similar trend (*F* = 4.22 *p* = 0.036). THKmax was positively correlated with SUVmax (*r* = 0.28, *p* = 0.004). SUVmax was higher but not significantly with AGC than MALT (*p* = 0.053).

**Table 1:** Incidence, intensity and thickness of gastric lesions in patients with PGL and AGC.

	ANHL(n = 24)	MALT (n = 14)	AGC (n = 69)
Presence of CT abnormalities	92% (22/24)	86% (12/14)	97% (67/69)
Presence of gastric FDGuptake	100% (24/24)	86% (12/14)	100% (69/69)
SUVmax	17.10±8.08*	6.20±3.60	9.65±5.24
THKmax (cm)	2.05±1.08	1.68±0.81	1.93±0.67

* SUVmax for ANHL vs. AGC and ANHL vs. FDG-avid MALT were significantly different by ANCOVA, *P*<0.05; SUVmax: maximum standardized uptake value; THKmax: maximal thickness of tumor; FDG: fluorodeoxyglucose; AGC: advanced gastric cancer; MALT: mucosa-associated lymphoid tissue; ANHL: aggressive non-Hodgkin’s lymphoma; Data are mean ± SD or number (%).

### CT-determined Abnormalities in Gastric Wall

**Figure 2:**
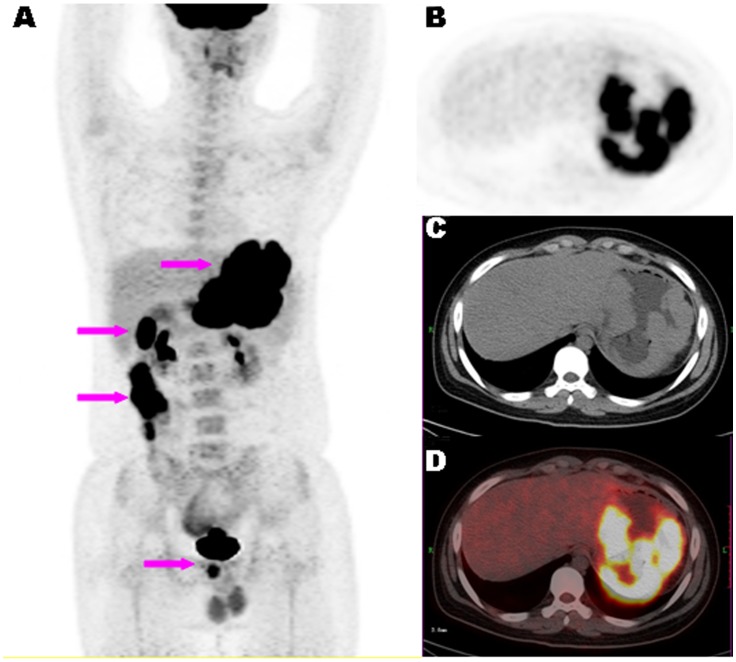
A representative case of ANHL . ^18^F-FDG PET/CT images of a15-year-old male patient with newly diagnosed Burkitt lymphoma. Maximum-intensity-projection view (A) showing multiple hypermetabolic lesions in the gastrointestinal tract (arrows). Axial PET (B), CT (C) and fused PET/CT (D) images showing diffused and irregular thickening of gastric wall with FDG uptake (SUVmax of 18.82).

Gastric CT-determined THKmax are also summarized in Table 1. THKmax did not differ among the 3 groups (*F* = 0.35, *p* = 0.56). A diffusely thickened gastric wall with multi-nodular intumesces was observed in only 1 patient with Burkitt lymphoma (Figure 2). Multiple lesions with segmental thickening of the gastrointestinal wall in the stomach and colon were observed in 1 patient with Burkitt lymphoma and 1 with diffuse large B-cell lymphoma. Ulceration was observed in 1 patient each of MALT and ANHL, and 9 with AGC; ulceration was seemed more common with AGC than PGL but the difference did not reach the significant level (*F* = 3.33, *p* = 0.32).

### Classifier for ANHL and AGC

**Figure 3:**
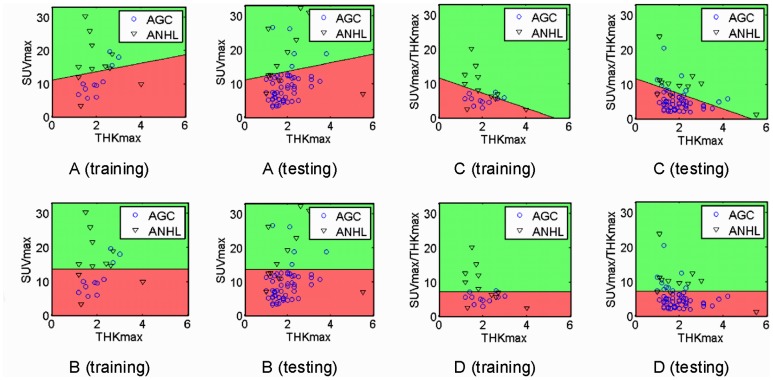
Linear classification analysis with SUVmax and CT-determined maximal thickness (THKmax) (A, training and testing), SUVmax alone (B, training and testing), normalized SUVmax (SUVmax/THKmax) and THKmax (C, training and testing) and SUVmax/THKmax alone (D, training and testing). Different colors represent the distribution of advanced gastric carcinoma (AGC) (red) and aggressive non-Hodgkin’s lymphoma (ANHL) (green) samples predicted by the classifier. AGC (ANHL) samples located in the red (green) region are correctly classified samples and those in the green (red) region are incorrectly classified samples. The THKmax value in plots of the bottom row (B and D) is for illustration only, not used for classification.

**Figure 4:**
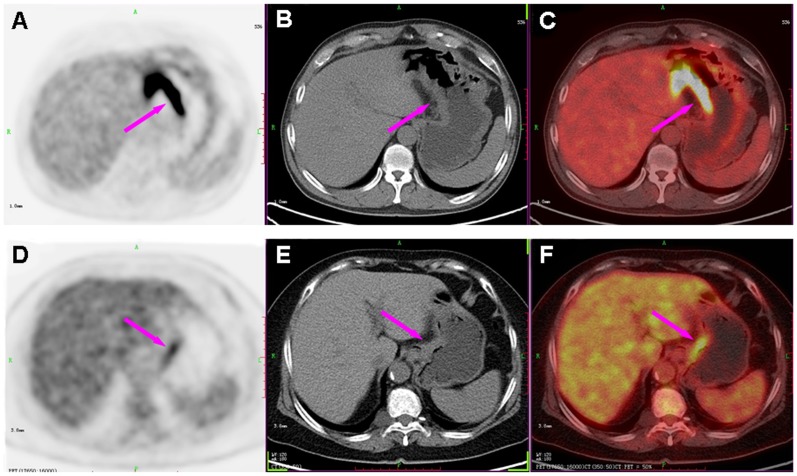
Comparison of PGL and AGC with representative cases. PET (left column), CT (middle column) and PET/CT fused images (right column) of a 56-year-old male with diffuse large B-cell lymphoma (A, B and C) and a 69-year-old female with poorly differentiated AGC (D, E and F), showing gastric lesions in the lesser curvature of stomach with similar thickness of the gastric wall (THKmax: 1.4 vs. 1.6 cm; arrows in B, E). However, the SUVmax was higher for PGL (SUVmax of 22.78; arrow in A) than AGC (SUVmax of 5.24; arrow in D).

The mean classification errors, sensitivity, specificity, 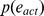, 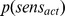
and 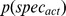 for different features are summarized in Table 2. Figure 3 demonstrates one implementation of the classification system with combined features. The mean SUVmax classification threshold value between ANHL and AGC was 13.40±1.12 and the mean SUVmax/THKmax was 7.51±0.63. As compared with SUVmax alone, the SUVmax/THKmax value improved the classification accuracy ((1-classification error) X100%) from 81% to 83%. Figure 4 shows the images for representative patients with PGL (diffuse large B-cell lymphoma) and poorly differentiated AGC. Despite sharing the similar THKmax values, the SUVmax and SUVmax/THKmax values were higher with PGL than AGC.

**Table 2:** The results of Fisher linear classifier and Permutation for different combinations of features.

	Classification error	Sensitivity	Specificity	*P* (e_act_)	*P* (sen_act_)	*P* (spec_act_)
SUVmax and THKmax	0.21 (0.05)	0.82 (0.07)	0.63 (0.16)	0.014 (0.03)	0.050 (0.07)	0.295 (0.20)
SUVmax only	0.19 (0.04)	0.85 (0.06)	0.63 (0.17)	0.002 (0.01)	0.002 (0.01)	0.333 (0.18)
SUVmax/THKmax and THKmax	0.20 (0.04)	0.83 (0.06)	0.66 (0.17)	0.007 (0.02)	0.040 (0.05)	0.260 (0.19)
SUVmax/THKmax only	0.17 (0.03)	0.86 (0.05)	0.65 (0.14)	0.0002 (0.0009)	0.0002 (0.0009)	0.321 (0.18)

Data are mean (SD); *P* (e_act_), *P* (sen_act_) and *P* (spec_act_) representing the mean *p*-value of the permutation test for the actual classification error, sensitivity and specificity.

## Discussion

The detection rate of early GC with FDG-PET was significantly lower for the diffuse than intestinal type [22], which may be due to the low expression of glucose transporter-1 in signet-ring cell carcinoma and mucinous adenocarcinoma [23]. However, in the present study, active FDG uptake was observed in all 3 cases of signet-ring carcinomas and 2 mucinous carcinomas in the advanced stage, which was consistent with the literature findings about the detection difference in AGC [24]. We postulate that the variable stages of the recruited cases even with the same pathological type might account for the divergence among different studies. Therefore, we did not further classify sub-groups of AGC to analyze FDG uptake. Our study have demonstrated a 97% detection rate of AGC with SUVmax 9.65±5.24, which was comparable to other findings [25], [26].

Because of the overlap of the SUVmax, statistical differences in FDG uptake among different malignancies at the group level may not provide the similar predictive information for each subject. We used linear classification analysis and permutation test technique to screen reliable parameters to distinguish ANHL and AGC at the individual level. In line with results from one-way ANOVA and ANCOVA, SUVmax was useful in differentiating both malignancies with reasonable accuracy, 81%. After normalization (i.e., SUVmax divided by THKmax [SUVmax/THKmax] to control the intercept from THKmax), the classification accuracy of SUVmax/THKmax was increased by 3% with the threshold 7.51±0.63. In addition, SUVmax/THKmax showed a highest sensitivity and a proper specificity among these 4 features in classification. Thus, the current convergent evidence indicates that SUVmax/THKmax may be used as a valid and practical biomarker in differentiation from AGC from ANHL. The remarkable advantage of this approach was to provide a concise but effective biomarker, only with two parameters (THKmax and SUVmax of the gastric lesions), to discriminate AGC from ANHL, which is easy to grasp and clinically useful, might be of great value for clinical radiologists. Although the accumulation of FDG in the stomach is thought to be of limited clinical significance in detecting gastric diseases, especially for early invasive GC and MALT [14], [27], the present data indicate that SUVmax to THKmax ratio may be a reliable biomarker in distinguishing, at least in part, ANHL and AGC. MALT has been claimed undetectable by FDG-PET [14], which was hypothesized to be related to the heterogenous cellular population and the low metabolic activity with the disease [28]; whilst, the emergence of foci of intense uptake in the low-grade lymphomas should raise suspicion of conversion to high-grade disease [29]. In the current study, except two MALT without abnormal FDG accumulation and CT findings, majority gastric MALT displayed mild FDG uptake in various macroscopic pattern. Unfortunately, such a relatively small sample restrained MALT to be included into our Fisher linear discriminant. Further researches may be needed to evaluate SUVmax/THKmax in differentiate MALT from non-MALT NHL or AGC.

We identified CT-detected abnormalities of FDG-avid lesions, including THKmax and the mucosal ulceration. THKmax had limited contribution to the differential diagnosis of gastric malignancies. In contrast, ulceration was fewer with PGL than AGC. In fact, as an epithelium derived malignancy, GC was commonly observed irregular surface of mucous membrane, even ulceration formation. However, PGL was derived from the submucosa and infiltrated beneath the mucous membrane, thus mucosal ulceration was relatively rare than that of GC [30]. However, the low presence of gastric ulceration detected by the CT component of PET/CT indicates that the presence of ulceration alone may not be enough to provide reliable differential diagnostic information to distinguish AGC from PGL. This finding might be due to the low-dose and non-enhancement CT scanning we used. Nevertheless, the morphological abnormalities could be used as additional variables to improve the FDG-PET assessment of gastric malignancies, which maybe due to the different biological behavior between two malignancies.

### Conclusion

The current study supports the value of SUVmax/THKmax as a valid FDG-PET/CT biomarker in distinguishing ANHL from AGC at individual level and of some promise for clinical application. Structural CT-detected abnormalities can provide additional information to improve the diagnostic performance of FDG-PET for assessing gastric malignancies. The real value of FDG-PET/CT in distinguishing AGC from PGL, especially ANHL, warrants further investigation.

### Limitations

Our study contains limitations. Our GC cases were at a late stage. The number of patients was not sufficient to confirm the real clinical value of SUVmax/THKmax in the differential diagnosis of AGC and PGL in other clinical settings. The relatively small samples of MALT lead to its exclusion from classification performance. The retrospective nature of the current study could not completely rule out biases in patient selection and image reading. Finally, we did not study the link between PET/CT findings and long-term results or the clinical impact on disease management. Therefore the results and conclusions drawn from the current study need to be verified by the further investigation.
